# A Latent Profile Analysis of Body Image and Disordered Eating Attitudes and Behaviors in Adolescent Boys

**DOI:** 10.1002/jad.70185

**Published:** 2026-05-28

**Authors:** Madeline R. Olwert, Shelley R. Hart, Delwin Carter, Natalie R. Stromberg

**Affiliations:** ^1^ Department of Human Ecology University of California Davis California USA; ^2^ Department of Child Development California State University Chico California USA; ^3^ Department of Mental Health Johns Hopkins University Bloomberg School of Public Health Baltimore Maryland USA; ^4^ Department of Education University of California Santa Barbara California USA; ^5^ Private Practice Chico California USA

## Abstract

**Introduction:**

Adolescence is a risk period for body dissatisfaction which can contribute to eating disorder development. Given sex or gender differences in body image and disordered eating, it is critical to examine body image and eating concerns in adolescent boys and whether mindfulness is associated with body image and eating attitudes and behaviors in this population.

**Methods:**

Self‐report questionnaires on body image, mindfulness, and disordered eating attitudes and behaviors were completed by 151 adolescent boys (*M*_*age*_ = 14.58 years, SD = 2.08; 51.7% White) from a rural western United States community. Body image patterns were evaluated utilizing latent profile analysis. Associations between body image, mindfulness, grade level, ethnicity, and disordered eating attitudes and behaviors were examined.

**Results:**

The analyses yielded a four‐profile model with “Satisfied” (52.1%), “Moderately Satisfied” (18.3%), “Satisfied & Weight Preoccupied” (15.5%), and “Unsatisfied & Weight Preoccupied” (14.1%) profiles. Profiles characterized by higher levels of overweight preoccupation showed more disordered eating attitudes and behaviors. Boys with greater mindfulness were more likely to belong to the “Satisfied” profile than the “Satisfied & Weight Preoccupied” profile.

**Discussion:**

Approximately 14% of the sample demonstrated clinically significant levels of disordered eating attitudes and behaviors, highlighting the need to screen adolescent boys for these issues. Multiple body image profiles were identified, with certain profiles being associated with greater disordered eating attitudes and behaviors. Adolescent boys with greater mindfulness were more likely to have body image satisfaction, indicating a potential utility in incorporating mindfulness practices in disordered eating preventions and interventions for male youth.

## 1 Introduction

Negative appraisals of one's body, or body dissatisfaction, is associated with poorer mental health and increased negative health behaviors and is a well‐established risk factor for eating disorders (McLean and Paxton 2019). Contradictory to the historical perception that eating disorders are “female issues,” eating disorder rates in men are increasing faster than rates in women (Gorrell and Murray 2019). Thus, it is imperative to understand eating disorder risk factors in the male population, such as manifestations of body dissatisfaction during adolescence, and potential protective factors to be leveraged in prevention and intervention efforts.

### 1.1 Adolescent Boys and Body Dissatisfaction

Adolescence is a risk period for negative body image due to biological, psychological, and social factors. Both girls and boys can experience increased self‐consciousness and social comparison during adolescence which contributes to body dissatisfaction (Mulgrew 2024). Adolescent girls are at risk for body dissatisfaction as they experience normal pubertal changes like increased adipose tissue deposits. This development moves girls further away from the thin female body type promoted as “ideal” by the media and in society (Mulgrew 2024). For adolescent boys, pubertal changes include increased muscle mass and shoulder width which may move them to closer to the “ideal” male body type (Ricciardelli 2012). However, experiencing later pubertal onset in comparison to peers can increase the likelihood of adolescent boys developing body dissatisfaction (Tarif et al. 2025).

For adolescent girls, greater body dissatisfaction has been shown to predict greater disordered eating attitudes and behaviors (Mustapic et al. 2015). Compared to boys and men, girls and women have historically received greater attention in body dissatisfaction and disordered eating research (Brown and Keel 2023). However, at subclinical levels, disordered eating attitudes and behaviors may be as common among men as women (National Eating Disorders Association 2022) and research has documented the association between body dissatisfaction and disordered eating in adolescent boys (Byrne et al. 2024). There has been substantial effort over the past several decades to examine the prevalence and precursors of eating disorders in the male population, resulting in an increased understanding of the similarities and differences between males and females regarding body dissatisfaction and disordered eating (Baker et al. 2019).

While women and girls may experience body dissatisfaction and, consequently, engage in disordered eating to reduce their overall body mass and achieve the “thin ideal” (Mulgrew 2024), the desires to appear thin or decrease body mass are not necessarily the drivers of body dissatisfaction and disordered eating attitudes and behaviors for men and boys. Particularly salient to men and boys is the drive for muscularity, which can push individuals to increase muscle mass and thus overall body mass (Lavender et al. 2017; Ricciardelli 2012). However, similar to women and girls seeking to become thinner, men and boys can experience the drive for leanness which results in behavior directed toward decreasing body fat (Mulgrew 2024; Ricciardelli 2012).

Weight stigma, anti‐fat bias, and fatphobia all refer to negative beliefs or attitudes toward bodies deviating from society's thin or lean ideals, making such stigma a male and female issue. Adolescents can be negatively impacted by weight stigma regardless of their actual body size (Juvonen et al. 2019), and weight stigma is associated with multiple negative outcomes for adolescents including increased mental health challenges, social and academic difficulties, body dissatisfaction, and disordered eating attitudes and behaviors (Puhl and Lessard 2020).

### 1.2 Mindfulness

Mindfulness involves a nonjudgmental and nonreactive awareness of one's thoughts, feelings, and bodily sensations (Greco et al. 2011). Possessing a nonjudgmental awareness of one's thoughts and feelings may be protective against the negative body appraisals and stigmas that contribute to body dissatisfaction and disordered eating attitudes and behaviors. Empirically, greater mindfulness has been associated with lower internalized weight stigma and less disordered eating in women (Fekete et al. 2021), and meta‐analytic evidence indicates that greater mindfulness is associated with lower levels of disordered eating (Sala et al. 2020). Emerging evidence in adolescents further suggests that mindfulness‐based interventions can improve body appreciation, reduce maladaptive eating behaviors, and enhance adaptive coping strategies (Kendirkıran and Bilgin 2024), although findings remain mixed and the literature is limited (Bacalhau et al. 2025). Notably, only two of the 70 studies included in Sala et al.'s (2020) meta‐analysis focused on adolescents, highlighting a critical gap in the literature. Further research is needed to elucidate how mindfulness relates to body dissatisfaction and disordered eating attitudes and behaviors in adolescent boys.

### 1.3 Current Study

The current study investigated different manifestations of body image among adolescent boys utilizing latent profile analysis. Latent profile analysis is a person‐centered approach that can capture heterogeneity in body image by identifying distinct subgroups of individuals based on patterns of multiple, co‐occurring factors. To our knowledge, prior body image research using latent profile analysis has relied on either fully adult (e.g., Jackson et al. 2022) or mixed sex (e.g., Hoffmann and Warschburger 2018) samples. Given that adolescent boys’ body image is multi‐faceted (Ricciardelli 2012), and that their body dissatisfaction is associated with disordered eating (Byrne et al. 2024), we sought to elucidate different body image patterns in adolescent boys and evaluate how such patterns differentially relate to disordered eating attitudes and behaviors.

Due to observed differences in body image and related concerns across racial, ethnic, and cultural groups (Ricciardelli et al. 2007), we examined whether body image profiles were associated with participant ethnicity. To explore differences in body image related to stage of adolescence, we tested whether grade level (middle school vs. high school) was associated with body image profiles. We also evaluated whether adolescent boys' mindfulness related to their body image profile. This was all done by including ethnicity, grade level, and mindfulness as covariates and modeling them as predictors of profile membership.

## 2 Methods

### 2.1 Participants and Procedure

The current study was secondary analysis of a subset of data from a larger study. For the current study, participants were extracted from the total sample of 420 youth, ages 11–18 (*M* = 14.54, SD = 2.07, 56.7% female), enrolled in local schools in a small, rural community in the western United States. The current sample included 151 adolescent boys ranging from 11 to 18 years in age (*M* = 14.58 years, SD = 2.08). Demographics were self‐reported (see Table 1).

**TABLE 1:** Sample demographics.

	Frequency	Percentage
Participants in middle school (6th–8th grade)	60	39.7%
Participants in high school (9th–12th grade)	90	59.6%
Parent not graduated from high school	21	13.9%
Parent with a high school diploma (w/wo any college)	65	43.0%
Parent with a college degree (BA, MA, & PhD)	57	37.7%
Participants from a dual parent home	107	70.9%
Participants from a single parent home	40	26.5%
Participants who identified as White	78	51.7%
Participants who belonged to an ethnic minority group	64	42.2%

Following parent and adolescent consent, adolescents completed self‐report questionnaires that were administered during class at participating schools over the course of several weeks and took approximately 45 min. Those not participating were offered an alternative activity. The study was approved by the Institutional Review Board of the California State University, Chico.

### 2.2 Measures

#### 2.2.1 Multidimensional Body‐Self Relations Questionnaire‐Appearance Scale

The 34‐item MBSRQ‐AS (Cash 2000) is a widely used instrument for body image assessment that has been validated with adolescent boys and girls as well as adults (Argyrides and Kkeli 2013; Roncero et al. 2015). The MBSRQ‐AS includes five scales: Appearance Evaluation (ω = 0.75; all values reflect the current sample), Appearance Orientation (ω = 0.83), Overweight‐Preoccupation (ω = 0.81), Self‐Classified Weight (ω = 0.84), and Body Areas Satisfaction (ω = 0.91). Anchors for these scales vary but all utilize a 5‐point Likert‐type scale. Overweight Preoccupation and Appearance Orientation were reverse scored so higher scores would consistently reflect healthier body image. The exception was Self‐Classified Weight where higher scores reflected more underweight and lower scores more overweight self‐perceptions. MBSRQ‐AS scale means were used as indicators in the latent profile analysis.

#### 2.2.2 Child and Adolescent Mindfulness Measure

The CAMM (Greco et al. 2011) is a 10‐item measure for youth (9+ years) that evaluates level of awareness during ongoing activities, as well as judgmental or avoidant responses pertaining to thoughts and feelings. Higher sum scores indicated greater mindfulness (0 [Never True] to 4 [Always True]; ω = 0.84). Mindfulness was incorporated into the model as a predictor of profile membership.

#### 2.2.3 Demographic Variables

Adolescents self‐reported their grade level. Participants in middle school (6th–8th grade) were coded as “0” and participants in high school (9th–12th grade) were coded as “1.” Adolescents also self‐reported their racial‐ethnic identity (White *n* = 78, Latino/Hispanic *n* = 13, African American/Black *n* = 5, Asian American *n* = 22, Native American *n* = 3, Biracial *n* = 21, Missing *n* = 9). Due to low sample sizes for specific ethnic groups, a binary variable was constructed for ethnicity; participants identifying as non‐Hispanic White were coded as “0” and participants who reported belonging to an ethnic minority group were coded as “1”. Grade level and ethnicity were incorporated into the model as a predictors of profile membership.

#### 2.2.4 Eating Attitudes Test

Disordered eating attitudes and behaviors were measured using the 18‐item version of the Eating Attitudes Test for adolescents (Maïano et al. 2013; McEnery et al. 2016). Lower scores indicate a healthier relationship with food and less eating psychopathology risk (1 [Never] to 6 [Always]). A cut score of 13 identifies youth with clinically significant disordered eating attitudes and behaviors (i.e., Sum of EAT‐18; McEnery et al. 2016). Several scales were not utilized in the current study. Fear of Getting Fat (ω = 0.79) was not included due to construct overlap with the indicator variable Overweight‐Preoccupation. Vomiting‐Purging Behavior (ω = 0.06) was not included due to low reliability. Exploratory analyses utilizing the omnibus Wald test indicated Social Pressure to Gain Weight (ω = 0.76) and Food Preoccupation (ω = 0.66) were non‐significant for the model. The outcomes retained for the final model were Eating‐Related Control (ω = 0.78; χ^2^ = 33.09, *p* < 0.001, LTB‐ω = 0.47), Eating‐Related Guilt (ω = 0.70; χ^2^ = 28.19, *p* < 0.001, LTB‐ω = 0.57), and Sum of EAT‐18 (χ^2^ = 30.42, *p* < 0.001, LTB‐ω = 3.15).

## 3 Analysis

Using Mplus v8.6 software (Muthén and Muthén 1998‐2021), a BCH three‐step (Asparouhov and Muthén 2021) latent profile analysis was conducted. Latent profile analysis is a person‐centered approach whereby response patterns on particular items (i.e., indicators—MBSRQ‐AS scale means) identify heterogeneity in a sample through statistical modeling. The latent profile analysis began with profile enumeration—starting with one profile and increasing the number of profiles until model fit no longer improved based on multiple goodness‐of‐fit statistics (Ferguson et al. 2019; Nylund‐Gibson and Choi 2018). Models were also evaluated for theoretical interpretation and other factors (e.g., size of the profiles).

Next, the BCH method (Asparouhov and Muthén 2021) was used to estimate error at the individual level to create weights in a separate file. This separate file was then used to estimate the final model with the addition of predictors of profile membership and distal outcomes. Relationships between predictors of profile membership and latent profiles were examined using multinomial logistic regression. These relationships were expressed on the logit metric with accompanying odds ratios (OR) and OR 95% confidence intervals (CI) to understand the meaningfulness of the relationships.

To evaluate outcomes, mean values were estimated for each profile. The relationships between outcomes and profiles were assessed by the omnibus Wald test, followed by an examination of its effect size (LTB‐ω; Lanza et al. 2013) based on the Cohen's *d* metric (Cohen 1992). If the Wald test was significant, then the LTB‐ω was reported and profile‐specific mean differences and effect sizes (95% CI) were examined for all pairwise comparisons (Asparouhov and Muthén 2014). Each association was evaluated with Cohen's *d* (1992) recommendation (i.e., *d* = 0.00, no effect; *d* = 0.20, small effect; *d* = 0.50, medium effect; and *d* ≥ 0.80, large effect).

### 3.1 Data Screening

Data was screened for missingness and normality. Four cases were missing data on all indicators and excluded from analyses. Indicator data demonstrated acceptable missingness (i.e., less than 5%; Tabachnick and Fidell 2007). Screening for skewness and kurtosis indicated acceptable levels (< 2.0). Finally, zero‐order item correlations were investigated, with several significant small‐to‐large correlations present (0.16 < *r* < 0.70).

### 3.2 Profile Enumeration

Fit statistics varied (see Table 2). The ABIC continued to decrease; however, the lowest BIC occurred with the four‐profile model. The VLMRT attained significance with a three‐profile model (signaling the two‐profile) and the BLRT become non‐significant with the seven (signaling the six‐profile). Both the *BF* and the *cmP* identified a four‐profile model as the best fitting. Entropy for the four‐profile model was 0.86 (acceptable; Celeux and Soromenho 1996) and the smallest profile in the four‐profile model was acceptable with 13% (Nylund‐Gibson and Choi 2018). Therefore, the four‐profile model was selected for interpretation.

**TABLE 2:** Fit indices for latent profile analysis models for adolescent boys.

Model	LL	BIC	ABIC	VLMRT *p*	BLRT *p*	*BF*	*cmP*
1	−919.64	1889.46	1857.80	—	—	0.00	0.00
2	−858.29	1796.86	1746.22	*0.00*	0.00	0.00	0.00
3	−835.29	1780.96	1711.33	0.10	0.00	0.03	0.03
4	−816.67	*1773.82*	1685.21	0.42	0.00	*9.13*	*0.88*
5	−803.83	1778.25	1670.64	0.21	0.00	93.86	0.10
6	−793.32	1787.33	1660.74	0.63	*0.00*	582.22	0.00
7	−784.64	1800.06	*1654.48*	0.27	0.67	—	0.00

*Note*: Italics indicates which model is supported by each fit statistic when appropriate.

Abbreviations: ABIC, adjusted BIC; BF, Bayes factor; BIC, Bayesian information criterion; BLRT, bootstrap likelihood ratio test; cmP, correct model probability; LL, Log likelihood; VLMRT, Vuong‐Lo‐Mendell‐Rubin test.

### 3.3 Profile Interpretation

**FIGURE 1:**
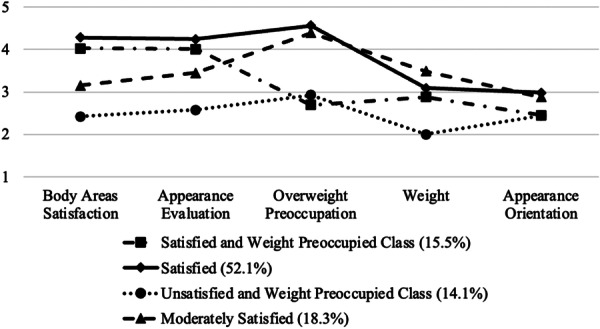
Conditional item probability plot for the four‐profile model of body image for adolescent boys. *Note:* Higher scores indicate healthier attitudes, with the exception of weight, with a “3” representing the healthiest attitudes, lower scores representing overweight and higher scores indicating underweight.

The profiles of the four‐profile model were distinct and interpretable (see Figure 1). The normative profile (*n* = 74, 52.1%; termed “Satisfied”) displayed high levels of satisfaction with body areas and appearance as well as low levels of overweight preoccupation relative to other profiles. This profile was further characterized by normal weight self‐classifications. A second profile (*n* = 22, 15.5%; “Satisfied & Weight Preoccupied”) also displayed normal weight self‐classifications and high levels of satisfaction with body areas and appearance. However, this profile had higher levels of overweight preoccupation compared to other profiles.

A third profile (*n* = 26, 18.3%; “Moderately Satisfied”) displayed moderate levels of body area and appearance satisfaction and lower overweight preoccupation relative to other profiles. Members of this profile were also more likely to report being somewhat underweight. The last profile (*n* = 20, 14.1% “Unsatisfied & Weight Preoccupied”) displayed low levels of body area and appearance satisfaction as well as high levels of overweight preoccupation. Members of this profile were also more likely to report being somewhat overweight.

### 3.4 Predicting Profile Membership

Ethnicity and mindfulness were used to predict profile membership via multinomial logistic regression. Members in the “Satisfied” profile were more likely to possess higher levels of mindfulness than those in the “Satisfied & Weight Preoccupied” profile (logit = 0.67, *p* = 0.023, OR = 1.96, OR 95% CI = 1.10, 3.49). This indicated that adolescents with higher levels of mindfulness were more likely to belong to the “Satisfied” profile than the “Satisfied & Weight Preoccupied” profile. The results for grade level and ethnicity were non‐significant.

### 3.5 Distal Outcomes

**FIGURE 2:**
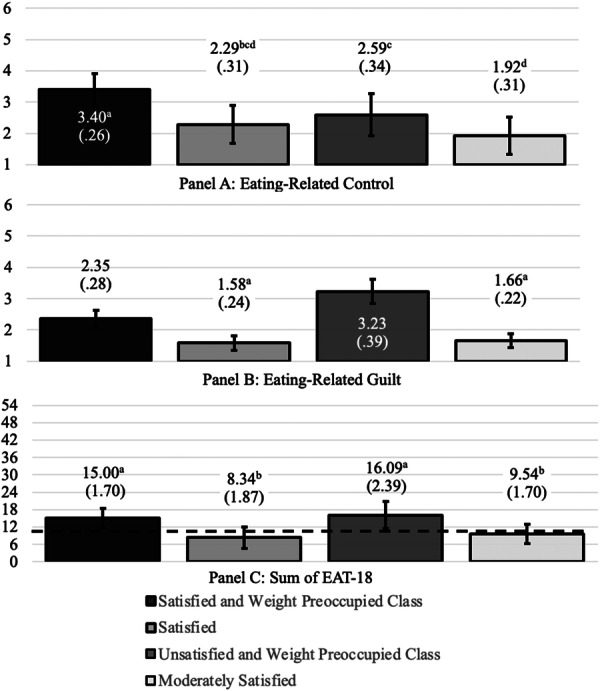
Means and standard errors for disordered eating attitudes by body image profiles. *Note*: Mean values that do not share a matching superscript letter indicate a statistically significant difference (*p* < 0.05). For example: in Panel C, the Satisfied and Weight Preoccupied class has a an “a” superscript while the Satisfied class has a “b” superscript and the Unsatisfied and Weight Preoccupied class has an “a” superscript. This indicates that the Satisfied and Weight Preoccupied class is significantly different than the Satisfied class but not significantly different from the Satisfied and Weight Preoccupied class. Dashed line indicates cut‐off value of 13 for Sum of Eat‐18.

Results for distal outcomes can be found in Figure 2, Panels A through C. Meaningful and statistically significant differences were found between profiles. For Eating‐Related Control (Panel A), those in the “Satisfied & Weight Preoccupied” profile had significantly higher levels than the “Satisfied” (*M*_*diff*_ = 1.12, *p* < 0.001, *d* = 1.35, 95% CI = 0.83, 1.85), “Unsatisfied & Weight Preoccupied” (*M*_*diff*_ = 0.81, *p* = 0.012, *d* = 0.95, 95% CI = 0.29, 1.58), and “Moderately Satisfied” (*M*_*diff*_ = 0.1.48, *p* < 0.001, *d* = 2.03, 95% CI = 1.33, 2.67) profiles. This indicated large effect sizes with scores for participants in the “Satisfied & Weight Preoccupied” profile ranging from 1 to 2 standard deviations higher than scores for participants in the other three profiles. Additionally, those in the “Unsatisfied & Weight Preoccupied” profile had higher levels than those in the “Moderately Satisfied” profile (*M*_*diff*_ = 0.67, *p* = 0.040, *d* = 0.75, 95% CI = 0.14, 1.34); a moderate effect with those in the “Unsatisfied & Weight Preoccupied” profile ¾ standard deviations higher than participants in the “Moderately Satisfied” profile.

For Eating‐Related Guilt (Panel B), those in the “Unsatisfied & Weight Preoccupied” profile had significantly higher levels than those in the “Satisfied” (*M*_*diff*_ = 0.77, *p* = 0.003, *d* = 2.29, 95% CI = 1.70, 2.85), “Satisfied & Weight Preoccupied” (*M*_*diff*_ = −.88, *p* = 0.043, *d* = 2.61, 95% CI = 1.99, 3.19) and “Moderately Satisfied” (*M*_*diff*_ = 0.70, *p* = 0.011, *d* = 2.07, 95% CI = 1.37, 2.71) profiles with large effect sizes of approximately two standard deviations. Participants in the “Satisfied & Weight Preoccupied” profile had significantly higher levels than the “Satisfied” (*M*_*diff*_ = 1.65, *p* < 0.001, *d* = 4.88, 95% CI = 3.97, 5.70) and “Moderately Satisfied” (*M*_*diff*_ = 1.58, *p* < 0.001, *d* = 4.65, 95% CI = 3.49, 5.66) profiles with very large effect sizes greater than four standard deviations.

For the Sum of Eat‐18 (Panel C), those in the “Satisfied & Weight Preoccupied” profile had significantly higher levels than the “Satisfied” (*M*_*diff*_ = 6.66, *p* < 0.001, *d* = 5.00, 95% CI = 4.10, 5.80) and “Moderately Satisfied” (*M*_*diff*_ = 5.47, *p* = 0.001, *d* = 4.12, 95% CI = 3.10, 5.01) profiles with very large effect sizes of 5 and 4 standard deviations, respectively. Participants in the “Unsatisfied & Weight Preoccupied” profile had significantly higher levels than the “Satisfied” (*M*_*diff*_ = 7.75, *p* < 0.001, *d* = 5.77, 95% CI = 4.74, 6.70) and the “Moderately Satisfied” (*M*_*diff*_ = 6.55, *p* = 0.005, *d* = 4.87, 95% CI = 3.67, 5.91) profiles with large effect sizes of approximately five standard deviations. Of interest, 13.9% of participants scored in the clinically significant range (i.e., ≥ 13) for disordered eating attitudes and behaviors.

## 4 Discussion

Our exploratory latent profile analysis of adolescent boys' body image identified four distinct body image profiles with meaningful associations with disordered eating attitudes and behaviors. The two profiles with the highest levels of overweight preoccupation (“Satisfied & Weight Preoccupied” and “Unsatisfied & Weight Preoccupied”) were both associated with problematic eating‐related control and eating‐related guilt despite the two profiles' distinct differences in appearance and body area satisfaction. Importantly, these profiles were significantly more likely to demonstrate clinically significant levels of disordered eating attitudes and behaviors (i.e., Sum of EAT‐18 ≥ 13; 4 to 5 STD higher than the “Satisfied” and “Moderately Satisfied” profiles). This suggests that overweight preoccupation is a contributing factor to disordered eating attitudes and behaviors in adolescent boys aligning with other work on weight stigma and adolescent health (Puhl and Lessard 2020). Practitioners should be cognizant of weight stigma when promoting health and addressing disordered eating behaviors in male youth.

There were no significant differences in outcomes between the “Moderately Satisfied” and “Satisfied” profiles. These profiles were characterized by lower levels of overweight preoccupation and showed lower levels of eating‐related control and eating‐related guilt. However, given that the “Moderately Satisfied” profile was characterized by lower body area satisfaction and appearance evaluation compared to the “Satisfied” profile, it is possible these profiles would differ on outcomes beyond the scope of this study such as self‐esteem and mental health. Additionally, while it is important to for male body image research to consider constructs other than muscularity and leanness (Barnes et al. 2020), including muscularity and leanness in the latent profile analysis indicators may have further differentiated profiles such as “Satisfied” and “Moderately Satisfied.”

The “Satisfied” profile had similar levels of satisfaction with overall appearance and specific body areas as well as self‐reported weight as the “Satisfied & Weight Preoccupied” profile yet demonstrated significantly lower levels of disordered eating attitudes and behaviors. These two profiles evidenced the only significant difference found regarding mindfulness, with adolescent boys with greater mindfulness being more likely to belong to the “Satisfied” profile than the “Satisfied & Weight Preoccupied” profile. This aligns with previous research on adults showing negative associations between mindfulness and eating disorder psychopathology (Sala et al. 2020) as well as mindfulness‐related constructs (e.g., self‐compassion) being protective against weight stigma and disordered eating (Fekete et al. 2021; Tylka et al. 2015). Therefore, greater mindfulness may be protective against body dissatisfaction as well as disordered eating attitudes and behaviors for adolescent boys. This study supports the incorporation of mindfulness concepts, particularly focused on weight preoccupation, into disordered eating attitudes and behaviors prevention and intervention programs for adolescents. Mindfulness‐based practices may promote adolescents’ self‐compassion during a period of heightened self‐awareness and social comparison.

## 5 Limitations and Future Directions

There are several limitations to note. This study relied on a small, convenience sample with approximately 53% of consents returned, thus limiting the generalizability of the results. We were unable to examine ethnic minority groups separately due to low sample sizes. Including ethnicity in the model as a binary variable (i.e., non‐Hispanic White vs. everyone else) may obscure meaningful differences between ethnic groups. Future research using latent profile analysis to study adolescent boys’ body image should strive for larger analytic samples with greater representation of multiple racial‐ethnic groups. Additionally, participants in our sample ranged from early to late adolescents but the dataset did not include direct measures of pubertal timing or physical maturation. Consequently, the potential associations between developmental stage and body image profiles could not be examined directly. Future research would be strengthened by objective measures of pubertal timing or physical maturation, as well as actual weight or BMI. Finally, although the MBSRQ‐AS has been validated with adolescent boys and captures information on multiple constructs pertaining to body image, it does not explicitly measure attitudes and drives toward muscularity and leanness. Future latent profile analyses on adolescent boy body image should include indicators pertaining to muscularity and leanness in addition to the constructs captured by the MBSRQ‐AS.

Despite the limitations noted above, we believe the findings of our exploratory latent profile analysis are an important addition to the literature. We accessed a diverse population (i.e., 39.7% of participants in middle school; 42.2% belonging to an ethnic minority group) from the region and identified distinct body image profiles with meaningful relations to disordered eating attitudes and behaviors in adolescent boys. Approximately 14% of our sample demonstrated clinically significant levels of disordered eating attitudes and behaviors, highlighting the need to screen adolescent boys for these problematic attitudes and behaviors. Our latent profile analysis suggests that there are multiple, distinct patterns of body image amongst adolescent boys, with certain patterns relating to greater disordered eating attitudes and behaviors. Importantly, these at‐risk male youth were identified through body image constructs outside of those typically investigated for boys and men (e.g., muscularity). Knowledge on disordered eating attitudes and behaviors and body dissatisfaction specific to adolescent boys would better prepare clinical and school practices to effectively prevent and treat disordered eating attitudes and behaviors at a critical stage in development.

## Author Contributions

**Madeline R. Olwert:** conceptualization, writing – original draft, methodology, writing – review and editing, formal analysis, visualization. **Shelley R. Hart:** conceptualization, methodology, writing – review and editing, formal analysis, writing – original draft, data curation; investigation, project administration, resources. **Delwin Carter:** methodology, formal analysis, writing – original draft, visualization, writing – review and editing. **Natalie R. Stromberg:** conceptualization, methodology, data curation, investigation, project administration, writing – original draft.

## Conflicts of Interest

The authors declare no conflicts of interest.

## Data Availability

The data that support the findings of this study are available on upon reasonable request. The data are not publicly available due to privacy or ethical restrictions.
